# Rupture spontanée de la rate chez un enfant

**DOI:** 10.11604/pamj.2019.32.184.4260

**Published:** 2019-04-16

**Authors:** Charifa Rachidi Alaoui, Mohamed Rami, Khalid Khatalla, Aziz Elmadi, Youssef Bouabdellah

**Affiliations:** 1 Service de Chirurgie Pédiatrique, CHU Hassan II, Fès, Maroc

**Keywords:** Rupture spontanée de la rate, douleurs abdominales, TDM, traitement conservateur, Spontaneous rupture of the spleen, abdominal pain, CT scan, conservative treatment

## Abstract

Les ruptures non traumatiques ou spontanées de la rate (RNTR) sont des entités rares mais potentiellement mortelles. La mortalité de cette affection est essentiellement liée au retard diagnostique et thérapeutique, ainsi qu'aux risques liés au terrain et à la gravité de la pathologie sous-jacente. Elles nécessitent dans la majorité des cas une splénectomie. Elles peuvent survenir soit sur une rate macroscopiquement saine, par exemple au cours d'une mononucléose infectieuse (MNI) ou d'un accès palustre ou sur une rate pathologique par exemple tumorale mai aussi dans certaines coagulopathies. Dans notre cas il s'agit d'un enfant de 6 ans suivi pour une coagulopathie admis pour douleurs abdominales diffuses brutales, une pâleur cutanéo-muqueuse avec un état hémodynamique stable. Le bilan biologique a révèle une anémie normochrome macrocytaire; le diagnostic de confirmation de la rupture était réalisé par l'échographie et le scanner abdominal. Vu la stabilité de l'état hémodynamique, la décision d'un traitement conservateur a été prise avec transfusions de 2 culots globulaires. L'évolution était favorable.

## Abstract (en)

Non traumatic or spontaneous splenic ruptures are rare but potentially fatal. Mortality is mainly due to delayed and therapeutic diagnosis as well as to the risks associated with a predisposed condition and with the severity of underlying pathologies. Splenectomy is necessary in the majority of cases. They can occur either in subject with macroscopically healthy spleen but, for instance, with infectious mononucleosis (IMN) or malaria or in subjects with pathologic spleen due to tumor, for example, but even in patients with some coagulopathies. We here report the case of a 6 year old child followed up for coagulopathy, admitted with diffuse violent abdominal pain, cutaneous-mucous paleness with hemodynamic stability. Laboratory tests showed macrocytic normochromic anemia; the diagnosis of splenic rupture was based on ultrasound and abdominal CT scan. Because the patient was hemodynamically stable, conservative treatment with 2 packed red blood cell transfusions was proposed. Patient’s outcome was favorable.

## Introductio

La rupture non traumatique de la rate est une entité très rare, qui engage le pronostic vital en l'absence de prise en charge adaptée et précoce. Le taux global de mortalité est estimé à 10-15% [1] vu que cette pathologie reste encore mal connue par la plupart des médecins devant un tableau d'abdomen aigu. Elle peut être idiopathique ou une complication d'une maladie infectieuse, néoplasique ou hématologique [2]. La majorité des cas rapportés dans la littérature sont secondaire à une infection surtout chez l'adulte. Nous rapportons le cas d'un enfant reçu aux urgences pour un abdomen aigu en dehors de tout traumatisme, nous allons présenter également une revue de la littérature actuelle sur cette entité rare.

## Patient et observation

**Figure 1:**
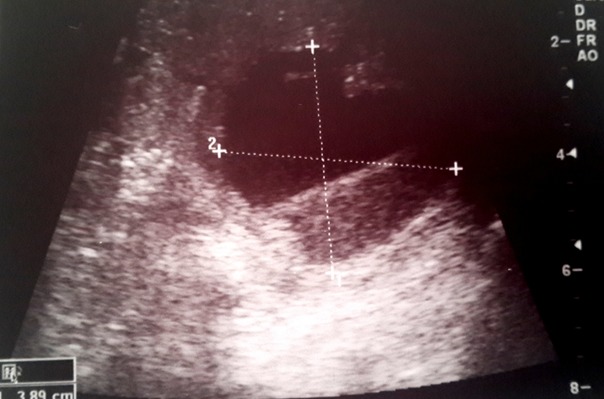
Coupe échographique objectivant en mediosplénique une image liquidienne à contours irrégulier et à contenu échogène

**Figure 2:**
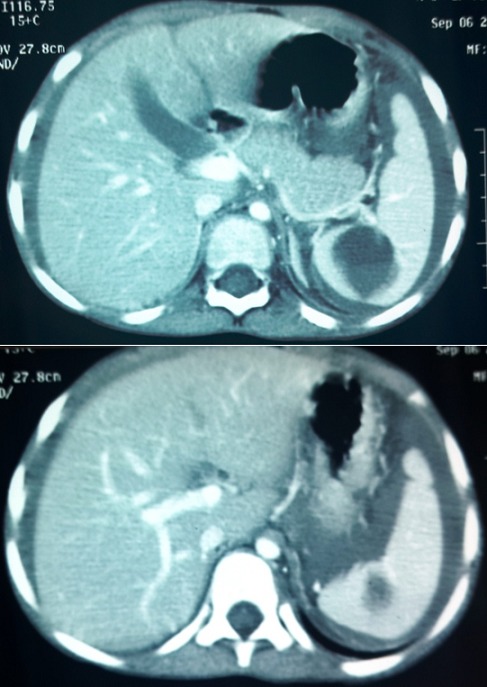
Coupe scannographique abdominale objectivant une fracture splénique avec un hémoperitoine de grande abundance

**Figure 3:**
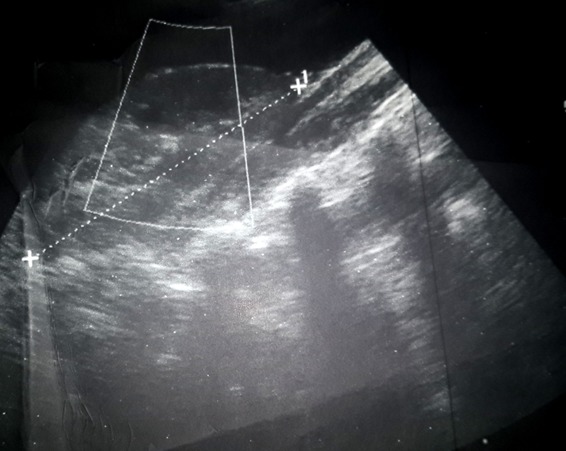
Contrôle échographique à 1 mois objective une régression de l’image mediosplénique

Il s'agit d'un enfant âgé de 6 ans sans antécédent (ATCD) pathologique notable ni notion de traumatisme, admis aux urgences pour un abdomen aigu. Le début de sa symptomatologie remontait à 2 jours avant son admission par des douleurs abdominales avec des vomissements sans trouble de transit, le tout évoluant dans un contexte d'Altération de l'état général (AEG) et d'apyrexie. A l'examen, il était apyrétique, une pâleur manifeste, tachycarde à 100, avec une TA 110/70 mm hg, une distension et une sensibilité abdominale généralisée. Le reste de l'examen est sans particularité. Le bilan lésionnel a objectivé une anémie à 6,6mg/dl avec, à l'abdomen sans préparation (ASP), une grisaille diffuse et à l'écho un épanchement de grande abondance avec une image hypoéchogène médio splénique (Figure 1). Un complément scannographique (Figure 2) a été réalisé confirmant le diagnostic d'une rupture splénique. Le traitement conservateur a été indiqué vu la stabilité hémodynamique de l'enfant avec transfusion de deux culots globulaires et une surveillance stricte en réanimation faite d'un monitorage de la tension artérielle et de la fréquence cardiaque avec la mise en place d'une voie centrale fémorale et une ligne artérielle. Un bilan étiologique a été réalisé notamment infectieux (goutte épaisse et la sérologie mononucléosique) revenu négative. Un bilan de coagulopathie et des hémopathies (TP.TQ. fibrinogène, bilan de drépanocytose, de la maladie de Gaucher et des lymphomes) a été lancé également revenant sans particularité. L'évolution a été favorable avec une amélioration clinique spectaculaire et stabilisation de l'état hémodynamique et biologique (HG à 12mg/dl), une amélioration radiologique au contrôle échographique (Figure 3). L'enfant a été déclaré sortant après un mois d'hospitalisation.

## Discussion

La rupture spontanée de la rate est une entité mal définie dont les causes favorisantes sont mal connues et le traitement reste controversé. Des auteurs suisses ont analysé 632 publications (845 malades) entre 1980 et concernant les adultes victimes d'une rupture spontanée de la rate; les ruptures spontanées survenant au cours d'explorations (coloscopie, échographie cardiaque trans-œsophagienne etc…) ont été exclues. Les ruptures spléniques spontanées ont été classées en 2 grandes catégories selon qu'une étiologie était retrouvée ou non. Aucune étiologie n'a été trouvée chez 59 sujets (7%); ce qui est le cas de notre malade; dont la rate était normale. Une étiologie a été mise en évidence chez 711 sujets (84%), deux étiologies chez 69 sujets (8%) et trois chez 6 autres (1%) par exemple: la tuberculose splénique, sarcoïdose et hémophagocytose). Les 3 étiologies les plus souvent en cause étaient les hémopathies (lymphomes non hodgkiniens), les maladies virales (mononucléose infectieuse -MNI-) et les inflammations de voisinage (pancréatites), qui ensemble étaient responsables de 42% des cas de rupture spontanée de la rate. A l'arrivée aux urgences, devant un tableau de douleurs et de choc hémorragique, l'hémopéritoine a été diagnostiqué par échographie, scanner, voire ponction dialyse péritonéale. Quant au diagnostic de rupture spontanée splénique, il a été porté lors de la laparotomie (42%), par le scanner (32%), l'échographie (19%), voire à l'autopsie (5%). Le diagnostic étiologique est une étape cruciale pour établir la conduite thérapeutique, c'était le cas chez 352 sujets (42%). Une splénomégalie a été signalée chez 79% des 591 malades pour lesquels la taille ou le poids de la rate ont été mentionnés (ce poids moyen étant alors de 700 g). Sur les 774 malades dont le traitement était précisé (exclusion des découvertes de rupture spontanée splénique à l'autopsie), 660 ont subi une intervention (651 splénectomies et 9 chirurgies conservatrices de la rate) avec 49 décès (7,4%). Les 114 autres ont subi un traitement médical (notamment pour MNI et paludisme), avec 16 splénectomies secondaires pour saignement itératif et 5 décès (4,4%).

Au total, 96 patients sont décédés (12,2%); les facteurs prédictifs de mortalité étaient les hémopathies, l'âge (> 40), la grosse rate. Si on n'a pu mettre en évidence l'influence du traitement, chirurgical ou conservateur sur la survie, on a noté une augmentation de la mortalité chez les malades opérés après un traitement initialement conservateur [3]. Au terme de cette étude qui regroupe une grande partie de cas rapportés dans la littérature le plus souvent sporadique on note que cette pathologie reste rare essentiellement chez l'enfant ou le nombre de cas rapporté est très limité; là on peut poser la question: est-ce vraiment une pathologie rare chez l'enfant ou sous diagnostiquée? Sur une autre étude bibliographique réalisée par Kianmanesh *et al.* après une recherche sur banques de données informatisées, 194 cas de RNTR publiés depuis les années 1960. Cette recherche révèle que les RNTR sont deux fois plus fréquentes chez les hommes. L'âge varie de 2 à 81ans (moyenne = 42ans). Il existe dans environ un tiers des cas des signes de choc lors du premier examen, comme cela était le cas pour notre malade. Dans 8% des cas, les malades décèdent avant d'être opérés et le diagnostic n'est fait qu'à l'autopsie. Dans 85% des cas, les malades sont traités par splénectomie [4]. Chez 7% des malades (surtout dans le cadre d'une MNI) un traitement médical conservateur sans chirurgie a été tenté [5-7]. Bien que cette analyse porte sur une période d'une quarantaine d'années, on peut estimer la mortalité postopératoire à 14%, ce qui confère aux RNTR une mortalité globale de 20% [8].

## Conclusion

En conclusion, les RNTR sont des entités rares, potentiellement mortelles et de diagnostic difficile. La mortalité globale est d'environ 20% et semble principalement liée au retard diagnostique et/ou à la gravité de la pathologie sous-jacente. Les étiologies infectieuses, dominées par la MNI et hématologiques, dominées par les hémopathies malignes, représentent à elles seules plus de la moitié des cas. Le diagnostic de RNTR doit être évoqué en cas de choc hémorragique associé à des douleurs abdominales en l'absence de tout traumatisme. Cependant, une rupture en deux temps est possible. Le diagnostic repose sur l'échographie ou la tomodensitométrie abdominale. Le traitement est la splénectomie, le traitement conservateur ne pouvant être proposé qu'à des malades sélectionnés ayant une MNI.

## Conflits d’intérêts

Les auteurs ne déclarent aucun conflit d'interêts.

## References

[1] Ayhan Y, Acar L, Erhan D (2010). Spontaneous rupture of spleen as a rare cause of abdominal pain: case report. Eur J Surg Sci.

[2] Leijnen M, Wobbe O, Brekelmans W, Da Costa A (2011). Non-traumatic rupture of the spleen: an Atypical presentation of the acute abdomen. Abdom Surg.

[3] Renzulli P, Hostettler A, Schoepfer AM, Gloor B, Candinas D (2009). Systematic review of a traumatic splenic rupture. Brit J Surg.

[4] Safapor F, Aghajanzade M, Kohsari MR, Hoda S, Safarpor D (2007). Spontaneous rupture of the spleen: a case report and review of the literature. The Saudi Jour of Gastroenterology.

[5] Kianmanesh R, Aguirre HI, Enjaume F, Valverde A, Brugière O, Vacher B (2003). Ruptures non traumatiques de la rate: trois nouveaux cas et revue de la literature. Annales de chirurgie.

[6] Asgari MM, Begos DG (1997). Spontaneous splenic rupture in infectious mononucleosis: a review. Yale J Biol Med.

[7] Conthe P, Cilleros CM, Urbeltz A, Escat J, Gilsanz C (1997). Spontaneous splenic rupture: surgical or conservative treatment?. An Med Interna.

[8] Johnson MA, Cooperberg PL, Boisvert J, Stoller JL, Winrob H (1981). Spontaneous splenic rupture in infectious mononucleosis: sonographic diagnosis and follow-up. AJR Am J Roentgenol.

